# Developing Interprofessional Primary Care Teams: Alumni Evaluation of the Department of Veterans Affairs Centers of Excellence in Primary Care Education Program

**DOI:** 10.1177/2382120519875455

**Published:** 2019-09-13

**Authors:** Nancy D Harada, Shruthi Rajashekara, Shubhada Sansgiry, Kathryn Wirtz Rugen, Samuel King, Stuart C Gilman, Jessica A Davila

**Affiliations:** 1 Centers of Excellence in Primary Care Education, Office of Academic Affiliations, U.S. Department of Veterans Affairs, Washington, DC, USA; 2 David Geffen School of Medicine, University of California, Los Angeles, Los Angeles, CA, USA; 3 Center for Innovations in Quality, Effectiveness and Safety, Michael E. DeBakey Veterans Affairs Medical Center, Houston, TX, USA; 4 Department of Medicine, Baylor College of Medicine, Houston, TX, USA; 5 College of Nursing, University of Illinois at Chicago, Chicago, IL, USA; 6 School of Medicine, University of California, Irvine, Irvine, CA, USA

**Keywords:** Interprofessional Education, Interprofessional Clinical Learning Environment, Veterans, Team-Based Care

## Abstract

**Purpose:**

The Centers of Excellence in Primary Care Education (CoEPCE) is an interprofessional graduate training program within the Department of Veterans Affairs (VA). In this project, we describe career paths of CoEPCE graduates, their perceptions of CoEPCE program value, their overall satisfaction with the training, and suggestions for program improvement to enhance interprofessional education and workforce development.

**Methods:**

The Graduate Participant Survey was developed and administered in 2018 to CoEPCE graduates from 2012 to 2017. Quantitative data from closed-ended questions were analyzed through descriptive and non-parametric statistics to test for significant differences by profession. Qualitative data from the single open-ended question were analyzed using content analysis with inductive and deductive approaches.

**Results:**

The survey was completed by 180 graduates. Greater proportions of pharmacists and psychologists than nurse practitioners and physicians were employed in VA, and greater proportions of nurse practitioners and pharmacists than physicians and psychologists were employed in primary care. Although smaller proportions of physicians were currently employed in primary care (*P* < .0001), a greater proportion completed advanced training programs (*P* < .0001). Overall, graduates perceived that their CoEPCE training was highly valued by advanced training programs and employers and improved their chances of finding a job. They reported high levels of satisfaction (mean = 4.3 ± 0.9 out of 5 total) with the training program, continued to use skills they learned during training, and believe their CoEPCE experiences made them better health care providers.

**Conclusions:**

Ninety-four percent of the CoEPCE graduates were employed at the VA and/or primary care at the completion of their training, although there were significant differences by profession. Graduates continued to practice interprofessional skills learned during their training and were highly satisfied with the program. Taken together, the findings indicate that continued enhancements to the interprofessional clinical learning environment are warranted.

## Introduction

Since 2010, policymakers have recognized the value of redesigning health professions education to develop a workforce capable of meeting the growing health care needs of the world’s population.^1-4^ Among their recommendations, a global commission proposed the need for interprofessional education that breaks down professional silos while enhancing collaborative and non-hierarchical relationships in effective teams.^1^ New instructional strategies focusing on interprofessional education would result in providers who are able to deliver collaborative, team-based care leading to enhanced efficiency, effectiveness, and improved patient outcomes.^2,5,6^

In 2010, the U.S. Department of Veterans Affairs (VA), Veterans Health Administration also began its work to develop new models of interprofessional education. The VA conducts the largest training effort for health professionals in the Nation accomplished through collaboration with 144 of the 152 accredited allopathic medical schools, all of the 34 accredited osteopathic medical schools, and other clinical health professions education programs such as nursing, psychology, and pharmacy.^7^ Administered through the VA Central Office, Office of Academic Affiliations (OAA), health professions education and training programs enhance the quality of care provided to Veterans and contribute to the development of the health care workforce for the VA and the Nation.

In 2011, OAA launched the Centers of Excellence in Primary Care Education (CoEPCE), a national multisite demonstration project to teach health care professions trainees to work in, lead, and improve patient-centered team-based care within primary care settings.^8-10^ The CoEPCE was established at the same time that the VA was implementing a new primary care delivery model based on the patient-centered medical home, which VA called patient-aligned care teams (PACT).^11,12^ Veterans Affairs’ introduction of PACT created the opportunity and necessity to develop new models of interprofessional education within these new VA primary care interprofessional clinical learning environments.^5,13^ There are 7 participating training sites across the United States. Five sites were initiated in 2011 (Boise, ID; Cleveland, OH; San Francisco, CA; Seattle, WA; and West Haven, CT) and 2 additional sites were initiated in 2016 (Houston, TX and Greater Los Angeles, CA). Mandated trainee professions include physician residents, nurse practitioner (NP) students and residents, pharmacy residents, and psychology fellows. Participation by other professions, such as social work interns and physical therapy residents, varies by site. New curricula were developed by interprofessional faculty to align 4 core educational domains (interprofessional collaboration, shared decision making, sustained relationships, and performance improvement) with teaching strategies for the aforementioned health care professions trainees.^9^ Interprofessional collaboration involves both team-based learning and care delivery; shared decision making focuses on communication between patients and providers and among team members; sustained relationships include connections between patients and providers and among faculty, staff, and trainees; and performance improvement includes both individual and team performance.^2,14^ All trainees received mentoring from interprofessional faculty and clinical staff in delivering team-based, patient-centered care.

Despite the call for interprofessional education as a way to address health care workforce shortages, there is limited information about the impact of these interprofessional education programs on future career decisions following program completion.^15,16^ In this article, we describe career paths of the CoEPCE graduates, their perceptions of CoEPCE program value, their overall satisfaction with the training, and suggestions for program improvement to enhance interprofessional education and workforce development.

## Methods

We conducted a cross-sectional survey of CoEPCE alumni who graduated between 2012 and 2017. This evaluation was categorized as an operation improvement activity based on Veterans Health Administration Handbook 1058.05, in which information generated is used for business operations and quality improvement. The overall project was subject to administrative oversight rather than oversight from a Human Subjects Institutional Review Board.

### Development of the Graduate Participant Survey

The CoEPCE Graduate Participant Survey (GPS) was developed by an interprofessional evaluation team composed of members from the CoEPCE national evaluation team and an external VA research advisory group located in Houston, TX, USA. Most items are closed-ended with 1 open-ended question at the end of the survey (Appendix 1). The closed-ended questions ask for demographic information, participation in advanced training programs, current employment, and perceived value of CoEPCE training to respondent’s advanced training program and/or employment. The instrument also asks respondents to rate, using 5-point Likert-type scales, the extent to which the CoEPCE training program achieved its mission to prepare graduates to work in and lead patient-centered interprofessional teams, as well as to rate other program impacts, including preparation for careers in primary care and opportunities for mentoring and precepting. Graduates indicated the frequency with which they practice skills in the 4 core educational domains in their current positions. The single open-ended question asks respondents to describe how the CoEPCE program could be improved.

GPS closed-ended questions were adapted from the CoEPCE Current Trainee Participant Survey, which was developed by the same evaluation team based on items adapted from the Interprofessional Education Collaborative (IPEC) to assess satisfaction with CoEPCE curriculum, program components, system impacts, and program practices.^17^ Extensive psychometric analysis of the Current Trainee Participant Survey has confirmed factor structure and internal consistency reliability with factor Cronbach’s alphas ranging from .85 to .96 (unpublished data).

### Data collection procedure

The GPS was administered in 2018 via SurveyMonkey over a 4-week period to graduates of the 7 sites. Sites were given the option to contact their graduates directly or to have graduates contacted by the evaluation team using the most recent email address on file. Two sites requested the email invitation come directly from the evaluation team, and 5 sites preferred to send email invitations themselves. The number of invitations sent out varied by site. The 5 sites initiated in 2011 had 6 years of graduates and sent between 54 and 162 invitations per site. The 2 sites initiated in 2016 had only 1 year of graduates and sent 5 invitations each. Across sites, a total of 475 graduates were contacted via email. The national CoEPCE evaluation team monitored response rates at all sites over the 4-week period. For those solicitations coming directly from the evaluation team, email reminders were sent to non-responders every 2 weeks. Sites that sent invitations to their own graduates followed up with non-responders directly. Response rates by site varied from 20% to 60%.

### Data analysis

Quantitative data from closed-ended questions were analyzed for all graduates and by profession. Descriptive statistics, chi-square tests for categorical data, and Kruskal-Wallis tests for continuous data were used to assess differences by profession. All quantitative analyses were conducted using SAS^®^ software, version 9.4 (SAS Institute Inc., Cary, NC, USA).^18^

Qualitative data from the single open-ended question asking how the program could improve were imported into Atlas.ti8.^19^ Content analysis using inductive and deductive approaches was used to assign open codes to responses and then group codes into themes based on GPS domains. Themes were discussed in relation to the quantitative findings by the full evaluation team to ensure representativeness across the sample.

## Results

A total of 180 graduates who completed training between 2012 and 2017 responded to the survey (40% response rate adjusted for unopened and non-working email addresses). The final sample of 180 graduates consisted of physicians (41%), NPs (29%), psychologists (19%), and pharmacists (11%). Graduates from all academic years were represented in the sample. The only significant difference across professions was in the amount of time spent in CoEPCE training (*P* < .0001). Fifty-one percent of all respondents spent 1 year or less in the program, comprised primarily of NPs, pharmacists, and psychologists. Thirty-nine percent (n = 70) of all respondents reported spending 2 or more years in the program, 81% (n = 57) of whom were physicians. These differences in time spent across professions were based on initial program design to accommodate differences in training models and duration across professions. Nurse practitioners could train in CoEPCE for up to 2 years as a part-time student and then a full-time resident, pharmacists and psychologists could train full-time in the CoEPCE for up to 1 year, and physicians rotated through the CoEPCE in block rotations throughout their 3-year residencies. These ambulatory blocks varied in duration and by site.

### Advanced training

One-third of the respondents across all professions (n = 60) reported participation in advanced training programs beyond the CoEPCE (Table 1). Physicians composed the highest proportion compared with other professions: 54% of the physician graduates chose advanced training compared with 21% of the NPs, 11% of the pharmacists, and 21% of the psychologists (*P* < .0001). Physicians continued to subspecialty fellowships in areas such as cardiology, nephrology, endocrinology, gastroenterology, and pulmonary critical care. Most of the graduates perceived value in the CoEPCE to their advanced training program. Of the physicians participating in advanced fellowship training, 65% felt that the advanced training program valued their CoEPCE training experience and 58% felt that the skills acquired during CoEPCE training improved their chances of getting into the advanced training program (Table 1).

**Table 1.** Advanced training: by profession (N = 180).

	Overall N (%)	Nurse practitioner N (%)	Pharmacist N (%)	Physician N (%)	Psychologist N (%)	*P*-value^a^
	N = 180	N = 53	N = 19	N = 74	N = 34	
Participated in advanced training (yes)	60 (33.3)	11 (20.8)	2 (10.5)	40 (54.1)	7 (20.6)	<.0001
Advanced training program values CoEPCE training experience (n = 60)^b^						.3261
To a great extent	22 (36.7)	6 (54.5)	1 (50.0)	12 (30.0)	3 (42.9)	
To some extent	23 (38.3)	5 (45.5)	0 (0)	14 (35.0)	4 (57.1)	
Not at all/not sure	15 (25.0)	0 (0)	1 (50.0)	14 (35.0)	0 (0)	
Skills acquired during CoEPCE training improved chances of getting into advanced training program (n = 60)^b^						.6807
To a great extent	14 (23.3)	4 (36.4)	0 (0)	7 (17.5)	3 (42.9)	
To some extent	24 (40.0)	4 (36.4)	1 (50.0)	16 (40.0)	3 (42.9)	
Not at all/not sure	22 (36.6)	3 (27.3)	1 (50.0)	17 (42.5)	1 (14.2)	

Abbreviation: CoEPCE, Centers of Excellence in Primary Care Education.

a Chi-square.

b n = 60 includes graduates who participated in advanced training.

### Employment

Nearly all CoEPCE graduates (94%) were currently employed in a paid position at the time of survey completion, ranging from 92% for NPs and physicians to 100% for pharmacists and psychologists (Table 2). Across all professions, 33% of the employed graduates reported current employment at the VA. Veterans Affairs employment differed significantly by profession: 68% of the pharmacists were employed by the VA compared with 50% of the psychologists, 25% of the NPs, and 21% of the physicians (*P* < .0001). Across all professions, 46% of the employed graduates reported current employment in primary care. Proportions of employed graduates working in primary care included 79% pharmacists, 63% NPs, 44% psychologists, and 25% physicians (*P* < .0001).

**Table 2.** Employment and perceived value: by profession (N = 180).

	Overall N (%)	Nurse practitioner N (%)	Pharmacist N (%)	Physician N (%)	Psychologists N (%)	*P*-value
	N = 180	N = 53	N = 19	N = 74	N = 34	
Employed in a paid position (yes)^a^	170 (94.4)	49 (92.5)	19 (100.0)	68 (91.9)	34 (100.0)	.2180
Roles in current position^a^						
Direct patient care	166 (92.2)	48 (90.6)	17 (89.5)	70 (94.6)	31 (91.2)	.7927
Educator	86 (47.8)	20 (37.7)	11 (57.9)	41 (55.4)	14 (41.2)	.1550
Evaluation/quality improvement	58 (32.2)	11(20.8)	12 (63.2)	20 (27.0)	15 (44.1)	.0022
Administration/management	44 (24.4)	11 (20.8)	5 (26.6)	16 (21.6)	12 (35.3)	.4052
Researcher	42 (23.3)	3 (5.7)	3 (15.8)	29 (39.2)	7 (20.6)	.0001
Likely to continue/seek future VA employment (mean ± SD)^b^	3.5 (±1.2)	3.7 (±1.2)	3.9 (±1.1)	3.1 (±1.2)	3.6 (±1.3)	.0302
Likely to continue/seek future primary care employment (mean ± SD)^b^	3.2 (±1.6)	3.7 (±1.4)	3.9 (±1.2)	2.4 (±1.6)	3.6 (±1.3)	<.0001
CoEPCE training improved chances of finding a job^a,c^						<.0001
Yes	108 (60.0)	42 (85.7)	14 (73.7)	25 (36.2)	27 (79.4)	
No	32 (17.8)	2 (4.1)	1 (5.3)	28 (40.6)	1 (2.9)	
Not sure	31 (17.2)	5 (10.2)	4 (21.1)	16 (23.2)	6 (17.7)	
Current employer values CoEPCE training^a,c^						.0004
To a great extent	48 (26.7)	18 (36.7)	5 (26.3)	12 (17.4)	13 (38.2)	
To some extent	73 (40.6)	19 (38.8)	13 (68.4)	25 (36.2)	16 (47.1)	
Not at all	24 (13.3)	3 (6.1)	0 (0.0)	20 (29.0)	1 (2.9)	
Not sure	26 (14.4)	9 (18.4)	1 (5.3)	12 (17.4)	4 (11.8)	

Abbreviations: CoEPCE, Centers of Excellence in Primary Care Education; VA, Veterans Affairs.

a Chi-square.

b Kruskal-Wallis test.

c Excludes missing (n = 9).

Respondents have multiple roles within their current positions. Across professions, 92% of the graduates provide direct patient care, 48% are educators, 32% are involved with evaluation/quality improvement, 24% with administration/management, and 23% with research. A higher proportion of physicians (39%) conduct research compared with other professions, but higher proportions of pharmacists (63%) and psychologists (44%) conduct performance improvement/evaluation compared with physicians (27%; *P* < .05).

The likelihood of graduates to continue or seek future employment in the VA (*P* = .0302) or in primary care (*P* < .0001) was statistically significant between professions. Pharmacists rated these items the highest at 3.9 (±1.1) and 3.9 (±1.2) on a 5-point scale with 5 the highest rating, and physicians rated each of these items the lowest at 3.1 (±1.2) and 2.4 (±1.6), for VA and primary care, respectively.

### Perceived value

Approximately 60% of all respondents reported that the CoEPCE training experience improved their chances of finding a job (Table 2). Although 86% of the NPs and 79% of the psychologists reported that their CoEPCE training experience improved their chances of finding a job, only 36% of the physicians felt this way, and 23% of the physicians were unsure (*P* < .0001). Professions differ significantly on perceptions of whether their current employers valued their CoEPCE training (*P* = .0004). Although 54% of the physician graduates felt their current employers valued their CoEPCE training to some or a great extent, they were the lowest compared with other professions.

Physicians continued to practice skills learned during their CoEPCE training in their current jobs (Table 3). Physicians’ rating on their practice of skills in interprofessional collaboration and in sustained relationships tended to be higher, although not significantly different from other professions (*P* > .05). The mean score on practice of performance improvement skills was low across all professions with the exception of pharmacists (*P* = .0279).

**Table 3.** Practicing skills learned during CoEPCE training in current jobs: by profession (N = 180).

	Overall Mean (±SD)	Nurse practitioner Mean (±SD)	Pharmacist Mean (±SD)	Physician Mean (±SD)	Psychologist Mean (±SD)	*P*-value^a^
	N = 180	N = 53	N = 19	N = 74	N = 34	
Interprofessional collaboration	4.3 (±0.7)	4.2 (±0.6)	4.3 (±0.6)	4.4 (±0.7)	4.2(±0.8)	.0510
Shared decision making	3.9 (±0.8)	3.9 (±0.8)	3.9 (±0.9)	4.0 (±0.8)	3.7 (±0.9)	.6214
Sustained relationships	4.0 (±0.9)	4.0 (±0.8)	3.9 (±1.0)	4.2 (±0.7)	3.6 (±1.2)	.0566
Performance improvement	3.4 (±0.9)	3.3 (±0.9)	4.0 (±0.8)	3.4 (±1.0)	3.4 (±0.8)	.0279

Abbreviation: CoEPCE, Centers of Excellence in Primary Care Education.

a Kruskal-Wallis test.

### Satisfaction and suggestions for improvement

Overall, on a 5-point scale, graduates reported that the CoEPCE had achieved its overall mission (4.0 ± 0.9), reported high levels of program impact (4.2 ± 0.7) related to primary care career preparation and mentoring/precepting, and were satisfied with the program (4.3 ± 0.9). Graduates believed that their continued practice of skills learned during CoEPCE training made them better health care providers. Suggestions for *program* improvement included better integration with professional training programs (eg, the larger internal medicine residency program), more opportunities for mentorship, and better communication and buy-in across the spectrum of facility leadership, faculty, and trainees. They also suggested a more explicit and structured curriculum. Suggestions for *curriculum* improvement related to the 4 core educational domains centered around increasing opportunities for practice, such as increasing representation and integration of different professions among the faculty and trainees for interprofessional collaboration; increasing opportunities for performance improvement, including through projects related to telehealth and health informatics; increasing communication and connections with other trainees and alumni for sustained relationships; and increasing opportunities to engage or communicate with patients through family meetings for shared decision making.

Finally, to better prepare trainees for careers in primary care, respondents requested guidance on preventing burnout and on transitioning from residency to primary care practice. Some of the graduates specifically mentioned wanting more information and opportunities related to finding work in the VA system.

## Discussion

Between 2012 and 2017, the CoEPCE developed new interprofessional curricula to train more than 700 unique health professions trainees to work in, lead, and improve team-based primary care. Through the GPS, we found that 94% of the responding graduates were currently employed in a paid position, 33% were employed by the VA, and 46% were employed in primary care. Compared with the other health professions, physicians were less likely to be retained in the VA or primary care, with only 25% of the physicians reported currently working in a primary care setting. Furthermore, we found that more than half of the physician graduates reported participating in another advanced training fellowship program after the CoEPCE.

Our physician’s rates of working in primary care are similar to those of West and Dupras who conducted an analysis of US internal medicine residents using an annual survey linked to the Internal Medicine In-Training Examination.^20^ Out of the 16,781 PGY3 residents, 21.5% reported career plans for practice in general internal medicine versus 64% who reported career plans for subspecialty practice. A notable difference is that our rates are specific to internal medicine physician residents who participated in the CoEPCE primary care interprofessional curriculum where one goal is to retain physicians to build the future health care workforce capable of providing interprofessional team-based care where ever they practice, for example, primary care or specialties, and therefore we would like to see our rates move higher. West and Dupras also found that between the PGY1 and PGY3 years, physician residents toggle between career plans in general internal medicine and subspecialty practice. They identified possible factors influencing career choice to be lifestyle considerations, anticipated income, match of future scope of practice with interests, and long-term relationships with patients.^20^

Future population projections highlight the growing aging population with complex health issues who will increase the demand for health care workers.^4^ In their study of physician retention in patient-centered medical homes in Canada, Ammi et al^16^ found that physicians were more likely to remain in primary care practices with more complex practice environments and greater numbers of vulnerable patients suggesting that physicians value serving those with the greatest needs. Other studies of primary care interprofessional collaborative practice models show that clinicians prefer to work in interprofessional teams.^21^

Through the CoEPCE we have developed new educational models to teach interprofessional skills to health professions trainees while improving the interprofessional clinical learning environments where the teaching takes place. However, to work effectively in these complex environments providers must possess interpersonal and technical skills to work effectively on interprofessional teams while maintaining professional identity and providing safe high quality care.^22^

Our findings suggest ways to increase the appeal of primary care practice for trainees across all health professions, generally falling into the following 3 categories: (1) increasing the value of team-based care delivery to providers through interprofessional education, (2) improving the interprofessional clinical learning environments where providers practice, and (3) expanding efforts to transition new graduates to these environments. With respect to the first category, we found that all professions valued team-based practice that could make them more effective providers. Higher proportions of physicians in our sample continued to practice skills in interprofessional collaboration, shared decision making, and sustained relationships after graduation compared with other professions after graduating from the CoEPCE. One physician commented that as the proximity of collaboration increased, there was more interaction and consulting with each other (other professions) leading to more integrated care for Veterans. We speculate that most physicians receive limited training in interprofessional care delivery during medical school. Physicians are also least likely to have exposure to other health care professional trainees prior to residency unlike other health professions where interprofessional training is part of the standard curriculum. Thus, learning these new skills and applying them during residency have potential to increase the personal and professional value placed on team-based care.

Continuing improvements to the interprofessional clinical learning environment will prepare health care providers to engage in safe and effective interprofessional collaborative care throughout their careers.^5^ In addition to incorporating the 4 educational domains of interprofessional collaboration, shared decision making, sustained relationships, and performance improvement, there is a need for strategies to address burnout in the primary care environment.^23,24^ Our findings confirm that burnout continues to be a concern among new graduates. Graduates’ suggestions for addressing professional longevity through interprofessional education and collaborative practice could include teaching interprofessional skills for more efficient and comprehensive patient examination and treatment, for example, shared visits and huddles or adjusting patient workloads based on team delivery models. The program improvements suggested by graduates also highlight the role of leadership in creating and sustaining collaborative environments.

Finally, to further encourage trainees to enter the primary care workforce, methods and guidance on transitioning from residency to primary care practice must be made available, including more information and opportunities related to finding work in community care and the VA. Among pharmacist and NP graduates, the impact of the CoEPCE program on developing the primary care workforce was significant. Seventy-nine percent of the pharmacists and 63% of the NPs were employed in primary care.

Pharmacists gave especially high scores to frequency of practicing skills in interprofessional collaboration and performance improvement in their current jobs. Nurse practitioners receive extensive training in interprofessional collaboration and patient-centered care in the formal graduate programs and therefore had training in these areas prior to the CoEPCE.

To our knowledge, this is the first survey to evaluate transition to practice of interprofessional trainees and to identify some of the methodological challenges in conducting follow-up surveys of graduate trainees from different health professions. Other studies of trainee follow-up using survey methodologies have been conducted on single professions targeting respondents remaining at the same training level so that contact information has not changed, for example, first- to third-year training,^25^ or have boosted their survey sampling frame by cross-referencing with a professional directory.^26^

Although the CoEPCE experience provides valuable insight on how to structure interprofessional education, there are some limitations. First, as the GPS was administered at 1 time point respondents differed in the time interval since graduation. Although we cannot determine whether bias played a role in survey participation, we know that respondents were distributed across all academic years and professions targeted for this survey. Second, we had contact information for 68% of the 700 CoEPCE graduates (n = 475) because some graduates did not leave updated personal contact information with their training sites to enable longitudinal follow-up. Finally, our final response rate of 40% was consistent to those response rates seen in other VA surveys.^27^

## Conclusions

Taken together, our findings lead us to conclude that interprofessional education in clinical learning environments has high potential to affect future health care workforce shortages with increasingly complex health care needs. CoEPCE graduates continued to work in the VA and/or primary care but these numbers can be increased by continuing improvements to the educational curriculum to teach health professionals to work effectively in teams within increasingly complex, high-stress clinical environments. Our work is just the beginning and we invite others to contribute to this work to further our understanding of how interprofessional education within an interprofessional clinical learning environment will meet the needs of all stakeholders including learners, patients, providers, policymakers, and populations.^2,28^

## Appendix 1: Graduate Participant Survey

### Centers of Excellence in Primary Care Education Graduate Participant Survey April 2018

**The purpose of this survey is to understand the impact (in your current work or training) of your experience in the VA Centers of Excellence in Primary Care Education (CoEPCE) training program. This will help us to improve training for future graduates. Your participation in this survey is greatly appreciated**.

### Centers of Excellence in Primary Care Education Graduate Participant Survey April 2018

#### Respondent Information

*1. Please indicate your former CoEPCE VA training site.
  - Boise
  - Cleveland
  - Greater Los Angeles
  - Houston
  - San Francisco
  - Seattle
  - West Haven
*2. When did you finish your training in the CoEPCE?
  - 2012
  - 2013
  - 2014
  - 2015
  - 2016
  - 2017
*3. Please indicate the amount of time you spent as a trainee in the CoEPCE.
  - Less than 6 months
  - 6-12 months
  - Greater than 1 but less than 2 years
  - At least 2 but less than 3 years
  - 3 or more years
*4. Please select your professional affiliation:
  - Nurse
  - Nurse Practitioner
  - Pharmacist
  - Physician
  - Psychologist
  - Social Worker

Other (please specify)

### Centers of Excellence in Primary Care Education Graduate Participant Survey April 2018

#### Advanced Training

*5. Have you participated in an advanced training program, such as a full-time fellowship or another graduate degree program since completing the CoEPCE training program?
  - Yes
  - No
*6. What is your current status in your advanced training program?
  - Currently enrolled
  - Completed
*7. What is the name of the advanced training program you are currently enrolled in or have completed?

*8. To what extent do you feel the advanced training program values your CoEPCE training experience?
  - Not at all
  - To some extent
  - To a great extent
  - Not sure
*9. Do you feel the skills you acquired during your CoEPCE training experience improved your chances of getting into an advanced training program?
  - Not at all
  - To some extent
  - To a great extent
  - Not sure

### Centers of Excellence in Primary Care Education Graduate Participant Survey April 2018

#### Employment

*10. Are you currently employed in a paid position?
  - Yes
  - No
*11. What is your current position title?

*12. Please select up to 3 activities that describe how you spend your time in your current position.
  □ Direct patient care
  □ Administration/ Management
  □ Educator
  □ Researcher
  □ Evaluation/Quality Improvement
  □ Other (please specify)

*13. Do you feel that your CoEPCE training experience improved your chances of finding a job?
  - Yes
  - No
  - Not sure
*14. To what extent do you feel your current employer values your CoEPCE training experience?
  - Not at all
  - To some extent
  - To a great extent
  - Not sure
*15. How much ongoing professional contact do you have with other trainees, alumni, or faculty from the CoEPCE training program?
  - Never
  - About once per year
  - 2-6 times per year
  - About once a month
  - Several times a month
*16. Are you currently employed by the VA?
  - Yes
  - No
*17. Are you currently employed in primary care?
  - Yes
  - No
*18. Have you ever been employed by the VA since completing your CoEPCE training?
  - Yes
  - No
*19. Have you ever been employed in a primary care setting since completing your CoEPCE training?
  - Yes
  - No
*20. How likely are you to continue or seek future employment in the VA?
  - Very unlikely
  - Unlikely
  - Neither likely or unlikely
  - Likely
  - Very likely
*21. How likely are you to continue or seek future employment in primary care?
  - Very unlikely
  - Unlikely
  - Neither likely or unlikely
  - Likely
  - Very likely

### Centers of Excellence in Primary Care Education Graduate Participant Survey April 2018

#### Mission and Program Achievements

**The overall mission of the Centers of Excellence in Primary Care Education (CoEPCE) is to “foster transformation of clinical education by preparing graduates of health professional programs to work in and lead patient-centered interprofessional teams that provide coordinated, longitudinal care.”**

*22. Based on your experience in the CoEPCE training program, to what extent do you feel the CoEPCE achieved the overall mission as stated above?
  - Not at all
  - Small extent
  - Moderate extent
  - Fairly great extent
  - Extremely great extent

### Centers of Excellence in Primary Care Education Graduate Participant Survey April 2018

#### Program Impacts

*23. How would you rate the following statements about the CoEPCE training program?

**Table:**

	Neither agree nor				
	Strongly disagree	Disagree	Disagree	Agree	Strongly agree
Mentored trainees to identify personal career goals.	◦	◦	◦	◦	◦
Prepared trainees for a career in primary care.	◦	◦	◦	◦	◦
Prepared trainees to practice collaboratively in teams to provide coordinated care.	◦	◦	◦	◦	◦
Provided opportunities for trainees to interact with preceptors from multiple professions.	◦	◦	◦	◦	◦

### Centers of Excellence in Primary Care Education Graduate Participant Survey April 2018

#### Interprofessional Collaboration

*24. Please rate the frequency with which you practice the following skills in your current role.

**Table:**

	Never	Rarely	Sometimes	Very Often	Always
Communicating with colleagues outside of my own discipline	◦	◦	◦	◦	◦
Providing clinical care as a member of an interprofessional team.	◦	◦	◦	◦	◦
Valuing the unique expertise of other health professionals.	◦	◦	◦	◦	◦
Communicating my roles and responsibilities clearly to other health professionals.	◦	◦	◦	◦	◦
Constructively managing conflict with other providers.	◦	◦	◦	◦	◦

### Centers of Excellence in Primary Care Education Graduate Participant Survey April 2018

#### Shared Decision-Making

*25. Please rate the frequency with which you practice the following skills in your current role.

**Table:**

	Never	Rarely	Sometimes	Very Often	Always
Partnering with patients in health care decision-making.	◦	◦	◦	◦	◦
Using motivational interviewing techniques with patients.	◦	◦	◦	◦	◦
Using information systems and telehealth technologies to facilitate communication to enhance patient-centered care.	◦	◦	◦	◦	◦
Engaging other health professionals in problem solving and shared accountability for health care outcomes.	◦	◦	◦	◦	◦
Constructively managing conflict with patients and families/caregivers.	◦	◦	◦	◦	◦

### Centers of Excellence in Primary Care Education Graduate Participant Survey April 2018

#### Sustained Relationships

*26. Please rate the frequency with which you practice the following skills in your current role.

**Table:**

	Never	Rarely	Sometimes	Very Often	Always
Facilitating warm hand offs to other providers.	◦	◦	◦	◦	◦
Developing trusting relationships with patients and families/caregivers.	◦	◦	◦	◦	◦
Communicating roles and responsibilities of team members clearly to patients and families/caregivers.	◦	◦	◦	◦	◦

### Centers of Excellence in Primary Care Education Graduate Participant Survey April 2018

#### Performance Improvement

*27. Please rate the frequency with which you practice the following skills in your current role.

**Table:**

	Never	Rarely	Sometimes	Very Often	Always
Collecting and analyzing data to assess performance improvement.	◦	◦	◦	◦	◦
Using performance improvement strategies to increase the efficacy of team-based care.	◦	◦	◦	◦	◦
Using information technology to manage patient panels.	◦	◦	◦	◦	◦
Using reflective practice to identify opportunities for improvement.	◦	◦	◦	◦	◦

### Centers of Excellence in Primary Care Education Graduate Participant Survey April 2018

#### Overall Satisfaction

*28. As you reflect back on your CoEPCE training experience, how would you rate your overall satisfaction with it?
  - Highly dissatisfied
  - Dissatisfied
  - Neither satisfied or dissatisfied
  - Satisfied
  - Highly satisfied

### Centers of Excellence in Primary Care Education Graduate Participant Survey April 2018

#### Suggestions for Improvement

*29. How could the CoEPCE training program improve?
